# Applications of Human Amniotic Membrane Patching Assisted Vitrectomy in the Management of Postoperative PVR in Complex Retinal Detachments

**DOI:** 10.3390/jcm12031137

**Published:** 2023-02-01

**Authors:** Tomaso Caporossi, Andrea Molle, Matteo Mario Carlà, Stefano Maria Picardi, Gloria Gambini, Alessandra Scampoli, Lorenzo Governatori, Patrizio Bernardinelli, Stanislao Rizzo

**Affiliations:** 1 Head, Neck and Sensory Organs Department, Catholic University of the Sacred Heart, 00168 Rome, Italy; 2 Vitreoretinal Surgery Unit, Fatebenefratelli Isola Tiberina Gemelli Isola Hospital, 00186 Rome, Italy; 3 Ophthalmology Unit, Fondazione Policlinico Universitario A. Gemelli IRCCS, 00168 Rome, Italy; 4 Ophthalmology Unit, ASST Melegnano e della Martesana, 20070 Vizzolo Predabissi, Italy; 5 Consiglio Nazionale delle Ricerche, Istituto di Neuroscienze, 56124 Pisa, Italy

**Keywords:** amniotic membrane, proliferative vitreoretinopathy, retinal detachment, vitreoretinal surgery, vitrectomy, retinal breaks, retinectomy, transforming growth factor beta, epithelial mesenchymal transition, new perspectives

## Abstract

Human amniotic membranes (hAMs) are extraembryonic tissues currently employed in the treatment of many ocular and systemic diseases. Several reports indicate that hAMs can suppress the signaling pathway of tissue growth factor beta (TGF-β), a cytokine that plays a major role in the pathogenesis of proliferative vitreoretinopathy (PVR) through the induction of epithelial-mesenchymal transition (EMT) in exposed retinal pigmented epithelium (RPE) cells. The present study was conducted to evaluate the efficacy of a modified vitrectomy procedure (hAMP-V) involving the extensive coverage of exposed RPE with hAM patches to prevent postoperative PVR in a series of 15 cases of retinal detachment complicated by severe preoperatory PVR. The primary outcome was to assess the rate of successful retinal reattachment of a single hAMP-V procedure at 6 months from silicone oil removal. Secondary outcomes included the collection of intraoperative data concerning the quantity, size, and scope of hAM patches, and the assessment of postoperative improvements in mean LogMar BCVA at 3 and 6 months. Successful retinal reattachment was obtained in 14 out of 15 eyes (93.3%). Surgical failure due to major recurrence of PVR occurred in 1 out of 15 eyes (6.7%). Postoperative improvements in mean LogMar BCVA were statistically significant (*p* < 0.05, paired *t*-test). No intraoperative and postoperative adverse effects were reported. The study helped to refine the surgical technique while also offering cues for future improvements.

## 1. Introduction

Proliferative vitreoretinopathy (PVR) is a multifactorial process that occurs as a complication in 5–10% of rhegmatogeneous retinal detachment cases [1], accounting for the most common cause of failure in vitreoretinal surgery. Surgical management of PVR includes scleral buckling and pars plana vitrectomy with extensive membranes dissection and peripheral retinectomy [2]. Long-lasting tamponade agents such as silicone oil and multiple interventions may be necessary to manage recurrences [3]. Results are often unsatisfactory with a success rate of 60–80% and worse outcomes as the disease severity increases [4]. Clinical factors associated with preoperative PVR include detachment extension and the presence of giant, large (wider than 3 disc diameters) and multiple retinal breaks [4,5,6]. Postoperative PVR is associated with intraocular hemorrhage during or following surgery, multiple interventions, extensive retinopexy by means of cryotherapy, diathermy or laser photocoagulation, and preoperative PVR [4,5,6,7,8,9]. Retinal pigmented epithelium (RPE) cells are multifunctional pluripotent cells playing a leading role in the pathogenesis of PVR. Mature RPE cells are mitotically quiescent under physiological conditions but can activate and proliferate when exposed to systemic factors resulting from the breakdown of the blood-retinal barrier following a retinal injury [10]. Activated RPE cells detach from Bruch’s membrane and lose their epithelial morphology in the process of epithelial–mesenchymal transition (EMT). The endpoint of EMT is the transformation of RPE cells into collagen-secreting fibroblasts and myofibroblasts that migrate into the vitreous cavity through retinal breaks. These cells along with components of microglia and activated macrophages can form contractile fibro-cellular membranes over the inner and outer surface of the neural retina, described respectively as epiretinal or subretinal PVR [11]. Additionally, a reactive process within the retinal tissue itself defined as intraretinal fibrosis can also occur [11]. Eventually, the contraction of fibro-cellular membranes leads to the formation of new retinal breaks, resulting in the recurrence of retinal detachment with further exposure and activation of RPE cells. Therefore, the development of PVR can be considered as a self-perpetuating and exaggerated cicatricial response to retinal injury. Transforming growth factor β (TGF-β) is a cytokine identified into three different isoforms in the posterior segment of the eye, namely TGF-β1, TGF-β2, and TGF-β3 [12,13,14,15]. Exposure of RPE cells to TGF-β leads to a down-regulation of epithelial markers [16,17,18,19], and an increase of mesenchymal markers (α-SMA and fibronectin) [20] and migration capacity. Conversely, the inhibition of TGF-β signaling prevents EMT of RPE cells both in vitro and in vivo [18,21,22,23]. High levels of TGF-β are also correlated with the contractile capability of hyalocyte-containing collagen gels [24], and the increase of TGF-β levels following treatment with intravitreal anti-vascular endothelial growth factor (anti-VEGF) agents is associated with the contraction of fibrovascular membranes in proliferative diabetic retinopathy and retinopathy of prematurity [25,26,27]. Amniotic membrane (AM) or amnion is a thin membrane located on the inner side of the fetal placenta, histologically composed by an epithelial layer, a basement membrane, and a layer of avascular mesenchymal tissue [28]. Human amniotic membranes (hAMs) are currently employed in the treatment of several ocular diseases due to their wound-healing, anti-inflammatory, and anti-fibrotic effects. The latter have been shown to improve the surgical outcomes of primary and secondary trabeculectomy procedures [29] and strabismus surgery [30,31]. In forniceal reconstruction surgery, hAMs constitute suitable replacement tissues to mucosal membrane transplantation [32,33], and are successfully employed in the treatment of advanced ocular cicatricial pemphigoid [34] and Steven Johnson Syndrome [35]. Although the clinical implications are not fully explored, several studies have confirmed that AMs can suppress the TGF-β pathway in a selected population of cells involved in the pathogenesis of PVR including fibroblasts, macrophages, and particularly RPE cells. Laboratory studies show how proliferation and production of TGF-β1 are inhibited in conjunctival fibroblasts grown over a substrate of AM [36]. Additionally, AMs noticeably suppress the transcript expression of TGF-β2, TGF-β3, and all isoforms of TGF-β receptors (TGF-βRs) in both pterygial and normal conjunctival fibroblasts cultures, resulting in a less mitogenic, less contractile, and less fibrogenic cellular phenotype [37]. Similarly, corneal limbal and stromal fibroblasts cultured on a cryopreserved AM scaffold show a suppression in the expression of all TGF-β isoforms and TGF-β type II receptor (TGF-βRII) transcripts [38]. Remarkably, a stronger suppression is observed when fibroblasts grow adjacent to the AM compared to when they are separated by a distance, suggesting that a direct contact with the extracellular matrix components of the AM is responsible for the greatest deal of the modulatory effect. Pro-inflammatory M1 macrophages cultured over sheets of AM undergo a switch in gene expression and protein secretion towards a less inflammatory phenotype with a distance-dependent gradient [39]. Notably, the presence of activated macrophages in the vitreous chamber is associated with an increased incidence of postoperative PVR [40]. Scaffolds of AM matrix increase the expression of functional markers (RPE65 and CD68) while decreasing EMT-related markers and inflammatory cytokines in overlying RPE cells [41,42]. Similarly, isolated matrix components such as purified Heavy Chain-Hyaluronan/Pentraxin (3HC-HA/PTX3) can suppress EMT, proliferation, migration, and contraction of cultured RPE cells [43]. In vitreoretinal surgery, the mechanical efficacy of hAMs in the restoration of retinal integrity has been demonstrated in a broad range of conditions including recurring, myopic or post-traumatic full-thickness macular holes [44,45,46], and posterior breaks involving the macula [47] or extending over patches of paravascular atrophy [48]. Recently, Rizzo, Caporossi et al. have successfully treated two cases of uncomplicated retinal detachment by positioning a patch of hAM under the neurosensory retina to seal peripheral breaks [49]. The procedures were carried out without the need for additional retinopexy owing to the adhesive nature of the hAM’s extracellular matrix and its capability to quickly attach to the RPE. The new technique was dubbed human amniotic membrane patching assisted vitrectomy (hAMP-V) to emphasize the pivotal role played by the hAM patches in achieving retinal reattachment. The present study was conducted to expand the potential of a modified hAMP-V procedure in the management of complex cases of retinal detachments with severe preoperative PVR. Specifically, we aimed to explore the ability of hAMs to coat the exposed RPE and seal retinal breaks while also evaluating the efficacy of their anti-fibrotic properties in the prevention of postoperative PVR.

## 2. Materials and Methods

The present investigation is a single center, interventional, non-randomized, unmasked prospective pilot study. Fifteen eyes of 15 patients were examined and treated between February 2021 and November 2022 at the Ophthalmology Unit of Fondazione Policlinico Universitario Agostino Gemelli IRCCS, Catholic University of the Sacred Heart, Rome, Italy. Inclusion criteria were the presence of rhegmatogenous or combined rhegmatogenous and tractional retinal detachment complicated by preoperative PVR of grade C or higher as defined by the Retina Society Terminology Committee classification [50]. Exclusion criteria were the presence of preoperative uveitis, endophthalmitis, unsealed penetrating or perforating globe injuries, and age < 18 years. All patients had preoperative pseudophakia and were almost always referred from other institutions for surgical intervention after previously unsuccessful vitreoretinal procedures, either failed or followed by recurrence. Prior to surgery, all patients received a comprehensive ophthalmologic examination including best-corrected LogMAR visual acuity measurement (LogMar BCVA), Goldman applanation tonometry (GAT), and dilated posterior segment examination by indirect ophthalmoscopy followed by additional investigation with eye ultrasound and spectral domain OCT (Solix Full-Range OCT, Optovue Inc, Freemont, CA, USA) if needed. Enrolled patients were scheduled for hAMP-V and the exact grading of preoperative PVR was assessed by a trained vitreoretinal surgeon (T.C.) and ultimately confirmed by intraoperative visualization. The main characteristics of the study population are reported in Table 1. All hAMP-V procedures were performed by the same surgeon (T.C.) under general or local anesthesia (peribulbar block). Following antisepsis with povidone-iodine solution all patients received a 23-gauge three-port vitrectomy with an additional port to accommodate for a 25-gauge chandelier probe (Constellation^®^ Vision System, Alcon Laboratories Inc, Fort Worth, TX, USA). Triamcinolone acetonide (Kenacort, Bristol Myers-Squibb, Princeton, NJ, USA) aided removal of residual vitreous body and was performed with careful polishing of the vitreous base by either shaving vitrectomy or cutting with a scissors probe. Extensive dissection of epiretinal membranes was carried out following the injection of a combined vital dye (Membraneblue-Dual^®^, DORC International, Zuidland, The Netherlands). Subretinal strains were removed with an end-gripping forceps through pre-existing or iatrogenic retinal breaks as needed. Persistent tractions were released by performing a peripheral retinectomy preceded by coagulation of the major retinal vessels with bipolar diathermy and followed by laser treatment of the margins. The detached retina was then flattened by injecting Perfluoro-N-Octane (EFTIAR Octane^®^, DORC International, Zuidland, The Netherlands) into the vitreous chamber. Amniotic membranes were trimmed into round patches of different sizes with a cutaneous punch (Disposable Biopsy Punch; Kai Medical, Solingen, Germany) and inserted into the vitreous chamber to be manipulated inside the perfluorocarbon liquid bubble. One or more patches were positioned as needed to seal posterior breaks; their borders embedded under the margins of the break without performing additional retinopexy, similarly to the plugging procedure described for macular holes [44,45,46]. Large peripheral breaks (wider than 3 disc diameters) were sealed with a patch of suitable size and prophylactic laser photocoagulation of the margins was then carried out. In all cases undergoing peripheral retinectomy one or more large grafts (diameter ≥ 6 mm) were positioned over the exposed RPE in the quadrant where preoperative proliferations were more severe or where their intraoperative removal was deemed to be less adequate. In the absence of significant preoperative or intraoperative differences among retinal quadrants, the patch would be applied inferiorly. When possible, multiple grafts were applied to obtain a wider coverage of exposed RPE in smaller retinectomy procedures involving three or less quadrants (Video S1). Finally, direct perfluorocarbon liquid to polydimethylsiloxane (PDMS) silicone oil (RS-OIL 1000 cSt, Alchimia Srl, Padua, Italy) exchange was performed followed by suturing of the scleral ports with absorbable threads. Cryopreserved hAMs required for hAMP-V were delivered from the eye bank of Lucca, Italy within 7 days from their intraoperative application. All patients were re-evaluated at postoperative week 1 and months 1 and 3 until scheduled for silicone oil tamponade removal. Silicone oil removal was performed by active aspiration (Constellation^®^ Vision System, Alcon Laboratories Inc, Fort Worth, TX, USA) and followed by a thorough staining with combined vital dyes (Membraneblue-Dual^®^, DORC International, Zuidland, The Netherlands) and intraoperative OCT (Rescan 700^®^, Carl Zeiss Meditec AG, Jena, Germany) visualization to identify any postoperative recurrence of PVR (Video S2). Further follow-up visits were then performed at postoperative week 1 and months 1, 3, and 6. The main outcome of the study was to assess the overall rate of successful retinal reattachment of a single hAMP-V procedure at 6 months from silicone oil removal. Successful cases of retinal reattachment were then further differentiated in “complete” and “partial” successes. Cases with minor or no postoperative PVR recurrence at the time of silicone oil removal, defined as the absence of fibrotic changes at the margins of retinectomies and retinal breaks, were considered a “complete success”. Cases with moderate postoperative recurrence of PVR needing partial surgical revision of the edges of the retinectomy or retinal breaks were deemed as a “partial success” if retinal reattachment at 6 months could still be obtained by means of short-lasting gas tamponade. Conversely, cases needing re-injection of silicone oil tamponade following extensive surgical revision would be registered as failed cases and exit the study to enter regular postoperative care. Secondary outcomes included the analysis of intraoperative data concerning the quantity and size of hAMs employed along with their surgical scope, the assessment of postoperative improvements in mean LogMar BCVA at 3 and 6 months from silicone oil removal, and the incidence rate of postoperative complications such as severe hypotony, elevated intraocular pressure (IOP), vitreous hemorrhage, endophthalmitis, and intraocular inflammation. The study received approval from the institution’s Ethics Committee and was conducted in accordance with the tenets of the declaration of Helsinki. Prior to enrollment, all patients provided their written informed consent.

## 3. Results

The study population included 13 out of 15 eyes with primarily rhegmatogenous retinal detachments (86.7%) including one case with a preoperative history of penetrating injury (6.7%), and 2 out of 15 eyes with combined tractional and rhegmatogenous retinal detachments from PDR (13.3%). The average number of attempts at retinal reattachment prior to enrollment was 1.6, with 9 out of 15 eyes receiving 1 procedure (60%), 4 out of 15 eyes receiving 2 procedures (26.6%), and 2 out of 15 eyes receiving 3 procedures (13.3%). Overall, a single hAMP-V procedure was successful in granting retinal reattachment at 6 months from silicone oil removal in 14 out of 15 eyes (93.3%). Complete success with minor PVR recurrence and non-fibrotic margins of retinectomies and retinal breaks was achieved in 13 out of 15 eyes (86.7%). The average duration of silicone oil tamponade was 93.2 days. Mean postoperative LogMAR BCVA at 3 months from silicone oil removal was 1.83, improving by −0.17 from baseline (*p* = 0.029, paired *t*-test); mean postoperative LogMAR BCVA at 6 months from silicone oil removal was 1.76, improving by −0.24 from baseline (*p* = 0.014, paired *t*-test). Preoperative conditions associated with worse visual outcomes included subretinal perfluorocarbon liquid bubbles in the perifoveal region in 2 out of 15 eyes (13.3%); a history of ischemic maculopathy from diabetic retinopathy in 2 out of 15 eyes (13.3%); iatrogenic wounding of the macula resulting in reactive gliosis in 1 out of 15 eyes (6.7%); and iatrogenic blunt trauma to the parafoveal region resulting in choroidal fibrosis in 1 out of 15 eyes (6.7%). Additionally, in all cases with posterior breaks (26.7%) there was macular involvement with irreversible photoreceptors loss. Preoperative optic atrophy was reported in 4 out of 15 eyes (26.7%). Preoperative hypotony, defined as IOP values < 6.5 mmHg was reported in 2 out of 15 eyes (13.3%) and resolved following silicone oil tamponade and successful retinal reattachment. Analysis of intraoperative data showed that a peripheral retinectomy was carried out on 11 out of 15 eyes (73%), of which 3 eyes received a full four-quadrants procedure, 4 eyes received a three-quadrants procedure, and 4 eyes received a two-quadrants procedure. On average, each patient would be implanted with 1.6 hAM patches (range 1–3), with a mean patch diameter of 5.25 mm (range 2–8 mm). The application of hAM grafts was used to manage posterior breaks in 4 out of 15 eyes (26.7%), peripheral large breaks in 3 out of 15 eyes (20%), and for each of the 11 eyes (73.3%) undergoing a retinectomy procedure at least one patch was applied to modulate fibrosis at its margins. No cases of postoperative endophthalmitis, vitreous or retinal hemorrhage, uveitis, hypotony, or ocular hypertension were reported. Outcome measures are summarized in Table 2.

## 4. Discussion

**Figure 1:**
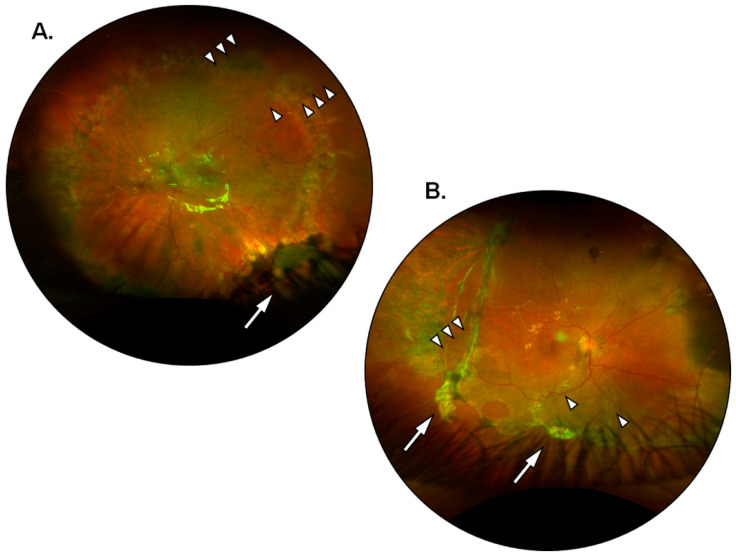
PVR recurrences following hAMP-V. (**A**) Fundus images of a partially successful hAMP-V showing a four-quadrant wide retinectomy with a large hAM patch applied adjacent to the inferior margin. The image was acquired at 1 month from surgery and shows initial recurrence of epiretinal PVR (multiple arrowheads) with a short subretinal strain (single arrowhead) over the superior margin of the retinectomy, distantly from the hAM patch. (**B**) Fundus image of a failed case of hAMP-V showing an extensive retinectomy of three quadrants with two hAM patches applied adjacent to its borders (arrows). The image was taken at 1 month from surgery and the patches appeared crumpled and opaque, with almost no ability to coat the exposed RPE. Recurrence of epiretinal PVR developed at the temporal margins of the retinectomy (multiple arrowheads) with subretinal strains radiating inferiorly from the optic disc (single arrowheads).

**Figure 2:**
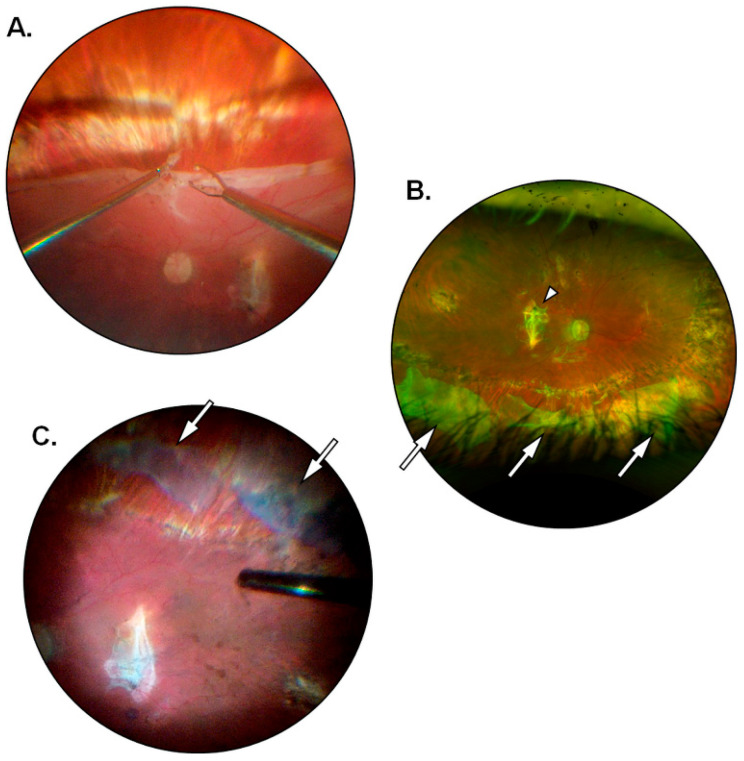
Management of a peripheral retinectomy with hAMP-V. (**A**) Intraoperative visualization of the surgeon performing an inferior peripheral retinectomy extending over three quadrants. (**B**) Postoperative fundus image at 1 month from hAMP-V showing three large hAM patches applied near the retinectomy margins (arrows) and coating a wide area of exposed EPR; the vitreous chamber is filled with silicone oil and a large macular scar can be seen (arrowhead). (**C**) Intraoperative visualization of the hAMs after silicone oil removal showing the non-fibrotic margins of the retinectomy with pigmented laser scars; the patches underwent minor folding of the edges and acquired a blue tinting due to the injection of vital dyes (arrows).

**Figure 3:**
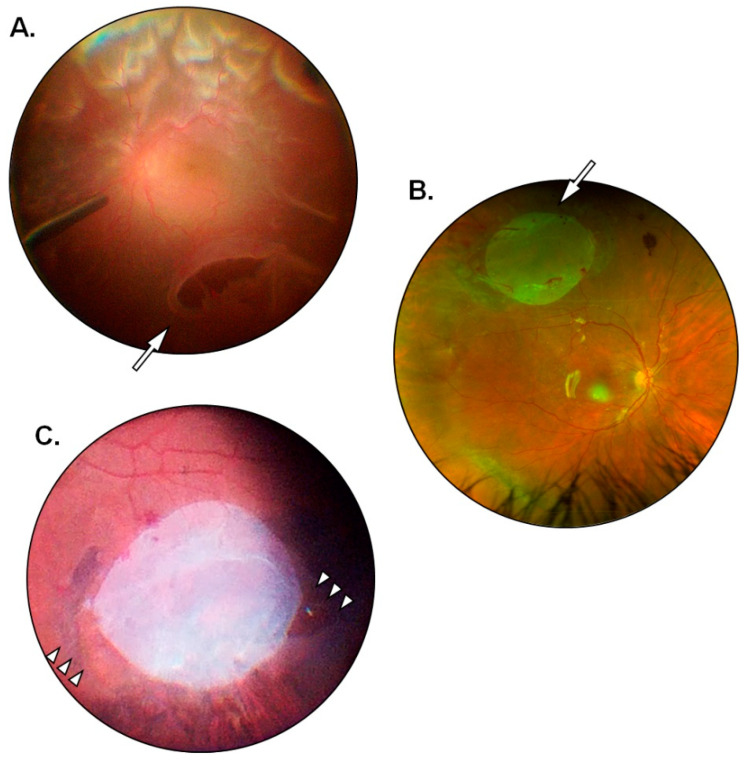
Management of a large peripheral break with hAMP-V. (**A**) Intraoperative visualization of a large peripheral break located superiorly (arrow). (**B**) Fundus image at 1 week from hAMP-V showing a large patch of hAM sealing the break (arrow) and coating the exposed EPR. (**C**) Intraoperative visualization of the hAM after silicone oil removal showing the non-fibrotic margins of the break surrounded by pigmented laser scars; the patch is leaving only minor sections of EPR exposed (arrowheads).

The present investigation provided insights on the behavior of hAMs in the modulation of postoperative PVR, particularly when the latter is observed in failed cases and cases of partial success. In 1 out of 15 eyes (6.7%) with preoperative grade D1 PVR that had received a four-quadrants retinectomy with the application of a single large hAM patch (diameter = 6 mm) a moderate recurrence of postoperative PVR occurred, encompassing the retinectomy margins and shortly extending into the subretinal space (Figure 1A). The case received partial surgical revision with a posterior recession of the retinectomy to remove the fibrotic borders and gain access to the subretinal strains. Successful retinal reattachment at 6 months was ultimately achieved following a short-lasting tamponade with a 10% mixture of air and sulfur hexafluoride (GOT SF6 Multi^®^, Alchimia Srl, Padua, Italy), thus accounting for a partial success. When managing the fibrosis at the retinectomy margins, in the absence of significant preoperative or interoperative differences, the patch was applied at the inferior quadrants as they correspond to the preferential localization of postoperative PVR following tamponade with PDMS. The uncommon, superior localization of PVR seems to be in accordance with the notion that the modulation of fibrosis is primarily contact and distance dependent. Moreover, a passive wash-out of soluble anti-fibrotic and immunomodulating factors including Tissue Inhibitors of Metalloproteinases (TIMPs), Interleukin-1 receptor antagonist (IL-Ira), and connective tissue growth factor (CTGF) has been reported to occur from the matrix of cryopreserved hAMs [51]. Perhaps, soluble factors released from the hAM are bound to gravitate inferiorly due to the displacing effect of silicone oil in the vitreous chamber; thus patches applied at the superior quadrants would still exert a degree of indirect, non-contact modulation over inferior PVR, but the opposite would not happen for inferiorly applied patches. Further improvements on the technique in patients undergoing four-quadrants retinectomy will involve the application of a single superiorly placed patch or multiple opposite-facing patches (i.e., superior plus inferior). The latter option would also provide a greater release of soluble mediators into the vitreous cavity. In 1 out of 15 eyes (6.7%) with a preoperative grade D3 PVR that had received a three-quadrants retinectomy with the implantation of two large hAM patches (diameter = 6 mm) a major PVR recurrence occurred along with posterior pole detachment and widespread subretinal proliferations. The patient needed extensive surgical revision and ultimately received long-term silicone oil tamponade, thus being registered as a failed case. In the latter, matrix adhesion was an issue, and membrane patches appeared crumpled and had lost their normal transparency shortly after application (Figure 1B). While the occurrence could be due to a defective or ill-preserved membrane, it draws attention to the lower stability of patches applied directly on the RPE without the presence of overlying retinal tissue to hold them in place. Future possible solutions would be to fit one edge of the hAM patch under the retinectomy margins or to apply a suitable mean of retinopexy onto the membrane itself. Nonetheless, in all other cases hAMs showed effective adhesion with only minor folding of the edges and would withstand the vacuum applied during active silicone oil aspiration to remain in place until the end of the study follow-up. The learning curve for hAMP-V was steep and it was not always viable to handle more than one large graft inside the vitreous chamber at the same time. However, with improved mastery of the technique over time, it was possible to obtain a broad deal of RPE coverage in smaller retinectomies involving up to three quadrants with excellent anti-fibrotic modulation of the margins (Figure 2). The sealing of retinal breaks proved to be efficient, and the hAMs would greatly reduce the area of exposed RPE, occasionally leaving minor uncovered sections near the edges of the break that did not result in PVR recurrence at 6 months from silicone oil removal (Figure 3). All hAMs embedded under the margins of retinal breaks remained in place for the entire duration of follow-up. It is reported how a partial re-growth of retinal layers can occur over a scaffolding hAM, both in the case of full-thickness macular holes and retinal breaks [48,52]. Given the difficulty to perform imaging on the study population, a systematic quantification of tissue re-growth is beyond the scope of this study. However, the reported regeneration of the neural retina, even if partial or non-functional, could have contributed to the stability of the hAMs sealing retinal breaks. As an additional benefit, hAMs applied to seal posterior retinal breaks allowed the surgeon to spare the retina from the damaging and pro-inflammatory effects of laser retinopexy. Future investigations will be aimed at testing the stability of hAMs sealing large peripheral retinal breaks without the aid of laser retinopexy, potentially reducing postoperative inflammation, and thus further enhancing the preventive effect on postoperative PVR. The loss of cell-to-cell contact seems to be responsible for the susceptibility of RPE cells to activating factors such as TGF-β. Studies on animal models show how activated RPE cells express adhesion molecules of mesenchymal lineage such as N-Cadherin and how the occurrence is reversible following retinal reattachment [53]. Since the loss of contact inhibition might be an important step in PVR development, its restoration alone through the apposition of scaffolding tissues could be effective in decreasing the pool of RPE cells available for migration and proliferation. The cutaneous punch allows for a round-shaped trimming of the hAMs before implantation; thus it is possible to estimate that on average each eye received the equivalent of 34.75 mm^2^ of RPE coating. For comparison, the latter amounts to a circular area almost 1 mm wider in diameter than a healthy macular region. As previously mentioned, a potential exists for hAMs to be employed as a source of sustained release of soluble anti-fibrotic and immunomodulating factors. Since the cellular components of hAMs are inactivated or destroyed following cryopreservation, soluble factors are not actively secreted but rather appear to be passively washed out from cryopreserved membranes [51]. Further studies are needed to assess the exact nature and amount of the factors being released, their timing, and their actual contribution to the prevention of postoperative PVR. However, it can be speculated that by applying a large surface of hAM patches a proportionally large quantity of soluble factors was also provided. The present study displays several limitations. Although statistically significant (*p* < 0.05), postoperative improvements in mean BCVA were poor as expected in eyes with a low baseline function and a history of multiple interventions. Additionally, most patients had preoperative conditions associated with unfavorable visual outcomes. To an extent, hAMP-V was employed in the study as a “rescue” treatment for complex cases with a history of failed surgical attempts at retinal reattachment. Nonetheless, with the aim of reducing the rate of surgical failures and reinterventions due to postoperative PVR, there is rationale to believe that an earlier application of hAMP-V in less complex cases could prove more beneficial in the improvement of postoperative BCVA. Postoperative recurrence of PVR occurred in eyes with rhegmatogenous detachments complicated by preoperative grade D PVR that underwent extensive three- or four-quadrants retinectomy procedures during hAMP-V. However, the sample of patients is too small to allow for a significant subgroup analysis. The small sample size could also have affected the results and thus a wider, multicentric population is needed to deal with confounding factors such as individual susceptibility, intraoperative variables, and different etiologies underlying the retinal detachment. It was not possible to fully standardize preoperatory variables other than the grade of preoperative PVR. Ultimately, patients with a different preoperative history were enrolled in consideration of the fact that the proliferative process would eventually reach the common endpoint of fibro-cellular membranes growth through the activation of the RPE cells. Limitations to the surgical technique include its rather steep learning curve and its reliance on the availability of a large amount of hAMs, which includes the need for facilities deputed to their adequate storage and preservation. In this study, hAMP-V was reserved for cases of retinal detachment scheduled to elective surgery, allowing for a demand-based supply of hAMs without any waste or leftovers. The high rate of success would reduce the number of reinterventions and thus effectively cover for the increased costs of hAMP-V compared to standard pars plana vitrectomy. To date, a substantial number of pharmacologic agents have been tested to reduce the development of postoperative PVR with often unsatisfactory results. Proposed treatments include anti-inflammatory agents [54,55,56,57,58], anti-mitotic drugs [59,60,61,62,63], or molecules with combined anti-metabolite and anti-inflammatory activity such as methotrexate [64,65,66,67,68,69]. The application of hAM patches is performed intraoperatively and does not require additional and repeated procedures on the patient, unlike most pharmacological treatments with intravitreal injections [64,67].

## 5. Conclusions

In our study, we proposed the addition of extensive hAM patching to pars plana vitrectomy in order to promote a broad contact between the exposed RPE and the extracellular matrix of the membranes, striving to restore contact inhibition, suppress EMT, and take advantage of their well-reported ability to restore retinal integrity. We employed hAMP-V to meet three distinct scenarios, namely the sealing of large retinal breaks, the coating of the RPE left exposed by retinal breaks and peripheral retinectomy and the anti-fibrotic modulation of their margins. We were able to achieve a high success rate in a small number of complex retinal detachments with severe preoperative PVR and a history of failed vitreoretinal procedures. The technique resulted in a low incidence of postoperative PVR at 6 months from silicone oil removal. To our belief, there is still room for future improvements in the application of hAMP-V, mainly in the choice of the number and positioning of hAM patches for cases undergoing four-quadrants peripheral retinectomy.

In conclusion, we found hAMP-V to be a new and promising approach in the management of challenging cases of retinal detachment with extensive preoperative PVR. Compared to pharmacologic agents designed to target a single molecular pathway, the modulation of EMT through hAMP-V would act on the common endpoint of the multiple factors concurring to the pathogenesis of PVR, while also providing mechanical and structural advantages within a single procedure. Further studies with a larger series of patients may confirm our results and lead to a broader employment of the technique.

## Figures and Tables

**Table 1:** Study population and preoperative data.

Population Study Characteristics	Value *
Number of patients	15
Mean Age (years)	60 ± 11
Male/Female ratio	9/6
Mean preoperative BCVA (LogMAR)	2 (2.70–1.50)
Mean number of Previous unsuccessful vitreoretinal procedures	1.6 (1–3)
Mean preoperative IOP (mmHg)	11.7 (5–19)
**Preoperative Epiretinal PVR, Grade D (eyes)**	**6**
Preoperative Epiretinal PVR, Grade D1 (eyes)	4
Preoperative Epiretinal PVR, Grade D3 (eyes)	2
**Preoperative Epiretinal PVR, Grade C (eyes)**	**9**
Preoperative Epiretinal PVR, Grade C2 (eyes)	5
Preoperative Epiretinal PVR, Grade C3 (eyes)	4
**Preoperative Subretinal PVR (eyes)**	**5**

BCVA = Best corrected visual acuity; IOP = intraocular pressure, PVR = proliferative vitreoretinopathy. * Value ranges are reported between commas.

**Table 2:** Outcome measures and intraoperative data.

**Study Outcomes**	Value *
Overall success rate (%)	93.3%
Cases achieving complete success (%)	86.7%
Cases achieving partial success (%)	6.7%
Failed cases (%)	6.7%
Mean hAM patches per patient (n°)	1.6
Mean hAM patch diameter per patient (mm)	5.25
Mean RPE coverage per patient (mm^2^)	34.75
Posterior breaks (eyes)	4
Peripheral large breaks (eyes)	3
Cases undergoing retinectomy (eyes)	11
Cases undergoing retinectomy, two quadrants (eyes)	4
Cases undergoing retinectomy, three quadrants (eyes)	4
Cases undergoing retinectomy, four quadrants (eyes)	3
Mean duration of silicone oil tamponade (days)	93.2 (85–102)
Mean IOP at 1 month postoperative, from hAMP-V (mmHg)	14.4 (10–17)
Mean IOP at 3 months postoperative, from hAMP-V (mmHg)	14.9 (12–17)
Mean BCVA at 3 months from silicone oil removal (LogMAR)	1.83 (2.70–1.00)
Mean BCVA at 6 months from silicone oil removal (LogMAR)	1.76 (2.70–1.00)
Mean IOP at 3 months postoperative, from silicone oil removal (mmHg)	12.4 (8–17)
Mean IOP at 6 months postoperative, from silicone oil removal (mmHg)	13.1 (9–16)

BCVA = Best-corrected visual acuity; hAM = human amniotic membrane; RPE = retinal pigmented epithelium; IOP = intraocular pressure. * Value ranges are reported between commas.

## Data Availability

The authors confirm that the data supporting the findings of this study are available within the article and its supplementary materials.

## Supplementary Materials

The following supporting information can be downloaded at: https://www.mdpi.com/article/10.3390/jcm12031137/s1, Video S1: Intraoperative footage showing the application of multiple hAM patches to manage a three-quadrants peripheral retinectomy, Video S2: Intraoperative footage showing active silicone oil removal, intraoperative OCT evaluation and staining with vital dyes at three months from hAMP-V.
